# Migraine Prevalence and Analysis of Dietary Habits in Relation to Headache in the Female Population: A Single-Center Study From Jeddah, Saudi Arabia

**DOI:** 10.7759/cureus.24848

**Published:** 2022-05-09

**Authors:** Yasser S Aladdin, Rawaf Alsharif, Weaam Mattar, Mona Alturki, Israa A Malli, Yara Alghamdi, Atheer Ismail, Bader Shirah

**Affiliations:** 1 Neurology Section, Department of Medicine, King Abdulaziz Medical City, Jeddah, SAU; 2 College of Medicine, King Saud bin Abdulaziz University for Health Sciences, Jeddah, SAU; 3 Research Office, King Abdullah International Medical Research Center, Jeddah, SAU; 4 College of Medicine, King Abdullah International Medical Research Center, Jeddah, SAU; 5 Basic Medical Sciences, King Abdullah International Medical Research Center, Jeddah, SAU; 6 Department of Neuroscience, King Faisal Specialist Hospital & Research Centre, Jeddah, SAU

**Keywords:** saudi arabia, headache, female, dietary habits, migraine

## Abstract

Introduction

Lifestyle-related factors including dietary intake can significantly increase the chances of developing migraine. Some components of food items are thought to induce the release of vasoactive substances leading to the dilation of blood vessels, which in turn results in migraine episodes. This research aimed to assess the prevalence of migraine and examine the relation of the headache to the dietary patterns of female students and employees of King Saud bin Abdulaziz University for Health Sciences (KSAU-HS) - Jeddah experiencing migraine attacks as well as to assess the relationship between the migraine attacks and the available dietary items served at the food outlets within the campus.

Methods

Two questionnaires were developed for this study. The first questionnaire was a short survey asking about the characteristics of headaches. This survey aimed to estimate the prevalence of migraine among female students and employees in KSAU-HS. Of the participants who filled the first questionnaire, those who met the International Classification of Headache Disorders (ICDH-III) criteria for migraine were given a second questionnaire to further understand the characteristics of their migraine headaches and to assess lifestyle and diet-related aggravating factors.

Results

The final sample size for the calculated prevalence was 410; 352 were students and 58 were faculty members. It has been found that 165 (40.24%) participants of the KSAU-HS female population met the criteria for migraine. Two (2.2%) reported an association between chocolate consumption and headaches and seven (7.8%) reported a significant association between caffeine and headaches. No specific association was perceived by the respondents between migraine attacks and the following food items: citrus fruits, pickles, cheese, and dairy products.

Conclusion

Our study revealed that the widespread trends for excessive consumption of coffee and caffeinated beverages at food outlets within the educational institution are occult triggers for headache attacks in a significant portion of students with migraine. The recent shift in dietary habits in our community for excessive consumption of coffee and other tyramine-rich food items has negative consequences on productivity and the economy. Our results can be conceivably extrapolated to reflect the effect of dietary habits on other streams of society, including companies, firms, schools, and workplaces that are driven by the new dietary trends.

## Introduction

Migraine is a headache disorder with specific characteristics and significant personal and socio-economic effects on the patient. According to the Global Burden of Diseases, Injuries, and Risk Factors studies in 2016, migraine ranked second globally among the leading causes of years of life lived with disability [1]. In Saudi Arabia, the prevalence of migraine is 26.9% with a male to female ratio of 1:2.9 [2]. The International Headache Society has developed specific diagnostic criteria for defining migraines. First, one episode of headache should last more than four continuous hours up to three days, with or without any successful treatment. Second, the headache should have two of the following characteristics: it should be either unilateral, pulsating, with severe or moderate intensity, or it should worsen with physical activities. Third, the headache should be accompanied by either nausea and vomiting or photophobia or phonophobia. Lastly, the headache should not be caused by pre-existing conditions or underlying disorders [3]. Several factors are thought to aggravate the headaches, including a disturbed sleeping pattern, emotional stress, hormonal imbalance, and dietary intake [4].

Migraine headache is a widespread disorder of recurrent pain affecting day-to-day life and can significantly impact the quality of life in any community, particularly in the young age group. One study of medical students with chronic headaches for more than a year revealed that about a third of them reported daily migraine attacks of increasing intensity resulting in fainting and neck stiffness. Significant triggers of their migraine included poor sleeping habits, changes in environment, sedentary lifestyle, and mental stress [5]. Therefore, factors related to lifestyle can significantly increase the chances of developing migraine with its consequences on productivity and life quality.

Another lifestyle-related factor contributing to migraine is dietary intake. Some components of food items, such as tyramine in cheese, phenylethylamine in chocolate, phenylalanine, caffeine, and food additives, such as aspartame, are thought to induce the release of vasoactive substances leading to the dilation of blood vessels, which in turn results in migraine episodes [4]. Aspartame, a synthetic sweetener, could influence the onset of migraine episodes in susceptible patients. In a randomized cross-over double-blinded study, aspartame was found to increase the frequency and severity of migraine in some of the subjects [6]. Another study reported that 19% of 490 patients suffering from migraine reported that the headaches were triggered by chocolate consumption, 18% by cheese, and 11% by citrus fruit. A high number of subjects reported sensitivity to all the foods mentioned. Around 31% percent of the 331 participating females reported that oral contraceptive use had negatively affected their headaches. Additionally, caffeine was recognized as a precipitating factor of a migraine but of moderate effect [7]. Although ingestion of specific types of food can induce migraine, skipping meals and dehydration were suggested to be factors that can influence migraine and need more research [8].

Migraine is becoming a serious public health concern as it is considered one of the leading causes of years lost to disability [1]. Chronic migraine has a significant impact on other aspects of daily life, including relationships, jobs, financial status, and overall well-being [9]. Moreover, migraine was found to be more debilitating among women, especially those of child-bearing age [10]. However, migraine prevalence has not been studied among females of the reproductive age in Jeddah. This research aimed to assess the prevalence of migraine and examine the relationship of the headache to the dietary patterns of female students and employees of King Saud bin Abdulaziz University for Health Sciences (KSAU-HS) - Jeddah with pre-existing migraine as well as to assess the relationship between the migraine attacks and the available dietary items served at the food outlets within the campus.

## Materials and methods

Two questionnaires were developed for this study. Each question in both questionnaires was typed in English and translated into Arabic. The first questionnaire was a short survey asking about the characteristics of headaches. This survey aimed to estimate the prevalence of migraine among female students and employees in KSAU-HS. Of the participants who filled the first questionnaire, those who met the International Classification of Headache Disorders (ICDH-III) criteria for migraine were given a second questionnaire to further understand the characteristics of their migraine headaches and to assess lifestyle and diet-related aggravating factors. In this study, a convenience sampling technique was used. Data were collected from participants by visiting their colleges. Participants were asked to sign an informed consent form before participating in the study.

Data acquisition

The first questionnaire was used as a quick screening tool for migraine. It was distributed by the research group members. It contained four direct questions. In the first question, the participants were asked about the duration of the headache. The second and third questions listed the features of headache, including the character of pain, aggravating factors, and associated symptoms. Participants were asked to check all the choices that apply. Lastly, participants who met the criteria for migraine were asked if they experienced any aura symptoms and were given the second questionnaire.

The second questionnaire starts with questions about demographic information. It includes three main sections. The first section contains questions for a general assessment of migraine. Questions about the average frequency of headaches per month, aggravating and alleviating factors, medication history, previous history of trauma, and the presence, type, and duration of aura were asked. In addition, previous diagnoses of other primary headache disorders and other health conditions were asked.

The second section contains questions about sleep quality and duration and the changing pattern of sleep habits during holidays and weekends and whether it had an effect on the frequency and severity of their headaches. The last section is about diet. In this section, participants were asked about their dietary habits. There were questions about particular food items that are believed to have an impact on migraine. Participants were asked about their consumption of the food item, and if they noticed it triggers a migraine episode. The food items included were: types of chocolate, dairy products, caffeine, citrus fruits, and pickles. Also, they were asked to mention any other food item that they noticed triggered their migraine episodes.

Analysis

Data analysis was performed using the SPSS Statistics v. 23 (IBM Corp., Armonk, NY). Frequency and percentages were used to display categorical variables, while mean and standard deviation were used to display continuous variables. The chi-square test was used to test for the presence of an association between categorical variables. ANOVA test was utilized to check the association between sleeping hours and frequency of headaches per month. The level of significance was set at 0.05.

Validation and ethical approval

The process of validation included a review of the questionnaire by three neurologists and then distributing the questionnaire to a sample of 31 participants. Ethical approval was obtained from the Institutional Review Board (IRB), King Abdullah International Medical Research Center (KAIMRC), Jeddah, Saudi Arabia.

## Results

The questionnaire was handed to 418 participants. Eight chose not to complete the questionnaire. The final sample size for the calculated prevalence was 410; 352 were students and 58 were faculty members at the College of Applied Medical Sciences (CAMS-J), College of Medicine (COM-J), College of Nursing (CON-J), and College of Science and Health Professions (COSHP-J). It has been found that 165 (40.24%) participants of the KSAU-HS female population have met the criteria for migraine.

Among the 352 students who responded to the questionnaire, 137 (38.9%) met the IHS criteria for migraine diagnosis. Among the 137 students who have migraine, only 48 students (35.04%) have an aura (Tables 1-2).

**Table 1:** Number and percentage of students who have migraine.

	Migraineur	Non-migraineur	Total number of students taken from each college
College of Science and Health Professions (COSHP-J)	65 (40.88%)	94 (59.12%)	159
College of Medicine (COM-J)	23 (41.07%)	33 (58.93%)	56
College of Nursing (CON-J)	34 (42.5%)	46 (57.5%)	80
College of Applied Medical Sciences (CAMS-J)	15 (26.32%)	42 (73.68%)	57
Total number of students from all colleges	137 (38.92%)	215 (61.08%)	352

**Table 2:** Number and percentage of students who have aura associated with their migraine.

	with aura	without aura	total number of migraineur students
College of Science and Health Professions (COSHP-J)	22 (33.85%)	43 (66.15%)	65
College of Medicine (COM-J)	5 (21.74%)	18 (78.26%)	23
College of Nursing (CON-J)	14 (41.18%)	20 (58.82%)	34
College of Applied Medical Sciences (CAMS-J)	7 (46.67%)	8 (53.33%)	15
Total number of migraineur students from all colleges	48 (35.04%)	89 (64.96%)	137

The total number of employees surveyed was 58. It has been found that 48.28% of the total number of female employees at KSAU-HS who completed the survey have met the IHS criteria for migraine (Table 3).

**Table 3:** Number and percentage of employees who have migraine.

	Migraineur	Non-Migraineur	Total number of employees taken from each college
College of Science and Health Professions (COSHP-J)	5 (27.78%)	13 (72.22%)	18
College of Medicine (COM-J)	9 (75%)	3 (25%)	12
College of Nursing (CON-J)	9 (45%)	11 (55%)	20
College of Applied Medical Sciences (CAMS-J)	5 (62.5%)	3 (37.5%)	8
Total number of students from all colleges	28 (48.28%)	30 (51.72%)	58

Among the 28 faculty members who met the criteria of migraine, 50% of them reported that they experience an aura phase (Table 4).

**Table 4:** Number and percentage of employees who have aura associated with their migraine.

	with aura	without aura	total number of migraineur employees
College of Science and Health Professions (COSHP-J)	2 (40%)	3 (60%)	5
College of Medicine (COM-J)	3 (33.33%)	6 (66.67%)	9
College of Nursing (CON-J)	6 (66.67%)	3 (33.33%)	9
College of Applied Medical Sciences (CAMS-J)	3 (60%)	2 (40%)	5
Total number of migraineur faculty members from all colleges	14 (50%)	14 (50%)	28

Of the 165 participants who met the criteria for migraine, only 90 returned the second questionnaire. Table 5 shows the sociodemographic profile of the participants. The mean age of participants was 22.4±5.7.

**Table 5:** Socio-demographic profile of participants (n = 90).

Demographical Characteristics	n	%
Age (mean, standard deviation)	22.4	5.7
Collage		
College of Medicine (COM-J)	32	35.6
College of Science and Health Professions (COSHP-J)	16	17.8
College of Applied Medical Sciences (CAMS-J)	21	23.3
College of Nursing (CON-J)	21	23.3
Nationality		
Saudi	89	98.9
Non-Saudi	1	1.1
Marital Status		
Single	84	93.3
Married	6	6.7
Occupation		
Student	84	93.3
Employee	6	6.7
BMI Class		
Underweight	16	17.8
Normal Weight	46	51.1
Overweight	15	16.7
Obesity Class 1	8	8.9
Obesity Class 2	1	1.1
Obesity Class 3	4	4.4

Table 6 displays the medical history of the participants. Only four (4.4%) were diagnosed with epilepsy or were taking anti-epileptic medications. Seven (7.8%) have once had a head or a neck injury requiring medical treatment. When asked if participants have ever been diagnosed with hypertension, asthma, heart disease, gastric ulcer, or dental problems such as temporomandibular joint, 21 (23.3%) said yes; 16 (17.8%) had sinusitis and five (5.6%) had deviated nasal septum.

**Table 6:** Medical history of participants (n = 90).

Question	n	%
Q1/ Have you been diagnosed with epilepsy/take any anti-epileptic medications?		
Yes	4	4.4
No	86	95.6
Q2/ Have you ever had a head or a neck injury requiring medical treatment?		
Yes	7	7.8
No	83	92.2
Q3/ Have you ever been diagnosed to have any health disorders (e.g. high blood pressure, asthma, heart disease, gastric ulcers, dental problems such as temporomandibular joint)?		
Yes	21	23.3
No	69	76.7
Q4/ Have you been diagnosed with one of the following?		
Sinusitis	16	17.8
Nasal polyps	0	0
Deviated nasal septum	5	5.6
None	69	76.7

Table 7 demonstrates participants’ responses toward frequency and types of headaches. The mean frequency of headaches was 5.67±5.32. Among the participants, only 13 (14.4%) had their headaches evaluated before. Among those who were evaluated before, 10 (11%) had migraine, two (2.2%) had both tension and migraine headaches, and one (1.1%) had a diagnosis other than headaches.

**Table 7:** Headache profile - frequency and type of headache (n = 90).

Question	n	%
Q1/ How many headaches do you experience per month on average?		
Mean	5.67	
Standard deviation	5.32	
Minimum	1	
Maximum	25	
Q2/ Have you had your headaches evaluated before?		
Yes	13	14.4
No	77	85.6
Q3/ If you had your headache evaluated before, what was the diagnosis?		
Tension headache	0	0.00
Migraine headache	10	11.
Both tension and migraine headache	2	2.2
Cluster headache	0	0
Other	1	1.1

Table 8 shows the participants’ attitudes and experiences with headache management. When asked if participants are taking any prescribed medication to treat headaches, only eight (8.9%) said yes. When asked if the participants are taking any over-the-counter medication, 72 (80%) said yes. As for the duration of time the headaches last after taking the medication, 59 (65.6%) reported no more than two hours, 23 (25.6%) reported three to four hours, five (5.6%) reported 5-12 hours, two (2.2%) reported several days, and one (1.1%) reported one week or longer. When asked how long the headache would last if a medication is not taken, 10 (11.1%) reported no more than two hours, 22 (24.4%) reported three to four hours, 28 (31.1%) reported 5-12 hours, 14 (15.6%) 12-24 hours, 14 (15.6%) reported several days, and two (2.2%) reported one week or longer. Also, 60 (66.7%) stated they were using painkillers in the past three months and 20 (22.2%) stated they were taking painkillers at least three times a week.

**Table 8:** Headache profile - management of headache (n = 90).

Question	n	%
Q1/ Are you taking any prescription medications to treat your headaches?		
Yes	8	8.9
No	82	91.1
Q2/ Are you taking any over-the-counter medications to treat your headaches?		
Yes	72	80
No	18	20
Q3/ How long do your headaches usually last after you take your medicine?		
No more than 2 hours	59	65.6
3-4 hours	23	25.6
5-12 hours	5	5.6
Several days	2	2.2
1 week or longer	1	1.1
Q4/ How long do your headaches usually last if you do not take your medicine?		
No more than 2 hours	10	11.1
3-4 hours	22	24.4
5-12 hours	28	31.1
12-24 hours	14	15.6
Several days	14	15.6
1 week or longer	2	2.2
Q5/ Have you been using any painkillers for the past 3 months?		
Yes	60	66.7
No	30	33.3
Q6/ Have you been using painkillers for at least 3 times per week?		
Yes	20	22.2
No	70	77.8

Table 9 displays the participants’ responses to questions surveying the severity and nature of headaches. When asked about the severity of headaches using a scale from 1-10, mild headaches between 1-3 were reported by 8.9%, moderate headaches from 4-6 were reported by 53.3% and severe headaches from 7-10 were reported by 37.8%. When participants were asked if headaches ever awaken them at night, 7.8% said often while 33.3% said occasionally. As for the location of headaches, 45 (50%) reported a frontal site, 28 (31.1%) reported an occipital site, 42 (46.7%) reported a behind eyes site, 33 (37%) reported both temples site, and 37 (41.1%) reported a one temple site. As for the nature of headaches pain, 69 (76.7%) reported a throbbing / pounding pain, 48 (53.3%) reported an aching / pressure pain, 21 (23.3%) reported a tight band pain, and 10 (11%) reported a dull pain. When asked if routine physical activity (walking or climbing stairs) can aggravate pain, 49 (54.4%) said yes.

**Table 9:** Headache profile - severity and nature of headache (n = 90).

Question	n	%
Q1/ How painful are your headaches (from a scale of 10)?		
1 - 3 (Mild)	8	8.9
4 - 6 (Moderate)	48	53.3
7 - 10 (Severe)	34	37.8
Q2/ Do your headaches awaken you at night?		
Never	53	58.9
Occasionally	30	33.3
Often	7	7.8
Q3/ Where are your headaches usually located?		
Frontal	45	50
Occipital	28	31.1
Behind the eyes	42	46.7
Both temples	33	37
One temple	37	41.1
Q4/ How would you describe your headaches?		
Throbbing / pounding	69	76.7
Ache / pressure	48	53.3
Like a tight band	21	23.3
Dull	10	11
Q5/ Does routine physical activity (e.g. walking or climbing stairs) aggravate the pain?		
Yes	49	54.4
No	41	45.6

Table 10 demonstrates the participants’ responses to questions about sleeping patterns. When asked how long they usually sleep, 45 (50%) reported a sleep duration of three to five hours, 38 (42.2%) reported a sleep duration of six to eight hours, and seven (7.8%) reported sleeping hours more than eight hours. As for sleeping duration during holidays, three (3.3%) reported a sleep duration of three to five hours, 34 (37.8%) reported a sleeping duration of six to eight hours, and 53 (58.9%) reported a sleeping hour of more than eight hours. When asked if participants noticed any change in their headache pattern on the weekends and during holidays, only 29 (32.2%) said yes.

**Table 10:** Sleeping patterns of the participants (n = 90).

Question	n	%
Q1/ How many hours do you usually sleep?		
3-5 hours	45	50
6-8 hours	38	42.2
More than 8 hours	7	7.8
Q2/ How many hours do you sleep over the weekends and holidays?		
3-5 hours	3	3.3
6-8 hours	34	37.8
More than 8 hours	53	58.9
Q3/ Have you noticed any changes in headache pattern in the weekends and during holidays?		
Yes	29	32.20
No	61	67.80

**Figure 1:**
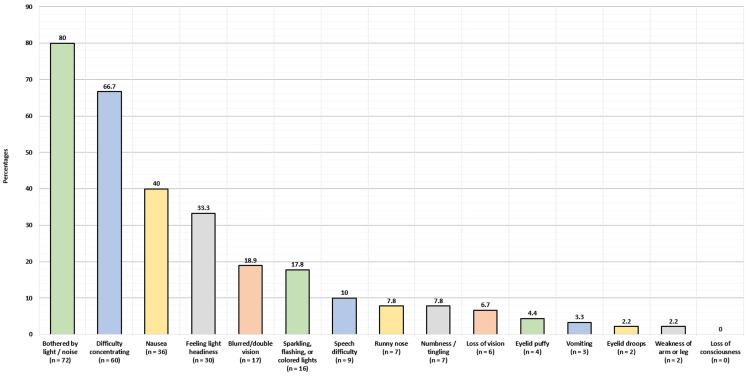
Signs and symptoms the participants experience during or before the headaches.

Figure 1 shows the signs and symptoms the participants experience during or before the headaches. The most commonly experienced symptoms were being bothered by noise and light (72; 80%), difficulty in concentrating (60; 66.7%), nausea (36; 40%), feeling light headiness (30; 33.3%), and blurred/double vision (17; 18.9%). The least commonly experienced symptoms were the weakness of arms (2; 2.2%) and eyelid drops (2; 2.2%).

**Figure 2:**
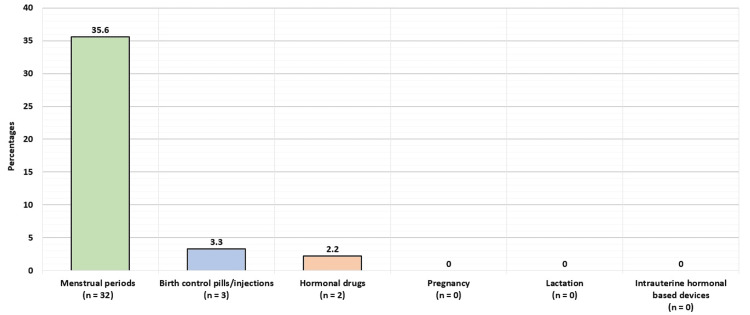
Participants’ gynecological factors associated with headaches.

Figure 2 displays the participants’ gynecological factors associated with headaches. Thirty-two (35.6%) reported menstrual periods to be associated with headaches, three (3.3%) reported birth control pills/injections, and two (2.2%) reported hormonal drugs. No association was reported between headaches and the following factors: pregnancy, lactation, and intrauterine hormonal-based devices.

**Figure 3:**
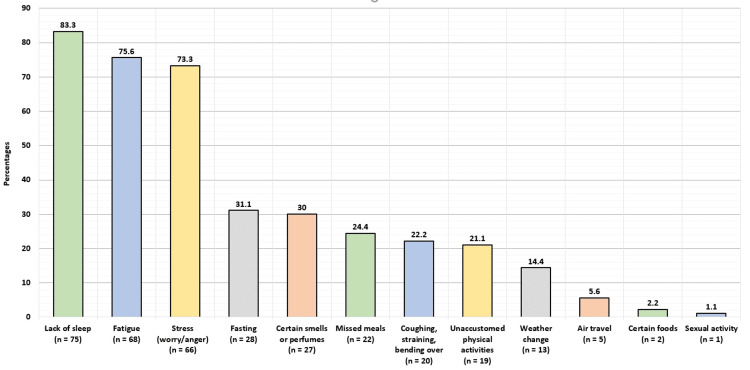
Factors reported by participants to trigger or worsen headaches.

Figure 3 demonstrates the factors reported by participants to trigger or worsen headaches. The most commonly reported factors were lack of sleep (75; 83.3%), fatigue (68; 75.6%), stress (66; 73.3%) (worry/anger), fasting (28; 31.1%), and certain smells or perfumes (27; 30%). The least commonly reported factors were sexual activity (1; 1.1%), certain foods (2; 2.2%), and air travel (5; 5.6%).

**Figure 4:**
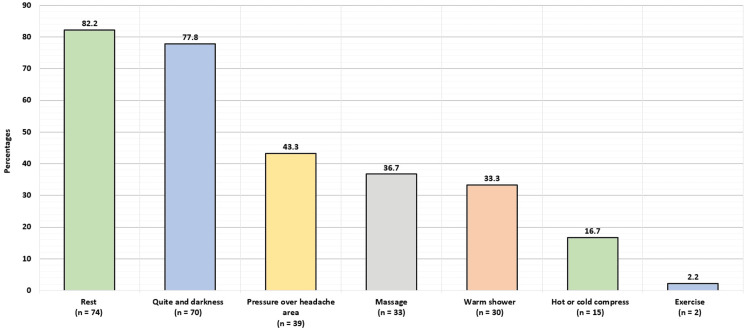
Factors reported by participants to relieve headaches.

Figure 4 shows the factors reported by participants to relieve headaches. The most commonly reported factors were rest (74; 82.2%), quiet and darkness (70; 77.8%), pressure over headache areas (39; 43.3%), and massage (33; 36.7%).

Table 11 displays participants’ dietary habits and their association with headaches. Two (2.2%) reported an association between chocolate consumption and headaches and seven (7.8%) reported a significant association between caffeine and headaches. No specific association was perceived by the respondents between migraine attacks and the following food items: citrus fruits, pickles, cheese, and dairy products.

**Table 11:** Dietary habits of participants and their association with headaches (n = 90).

Question	n	%
Chocolate		
Q1/ How often do you consume chocolate per week?		
1-3 times per week	55	61.1
5-10 times a week	13	14.4
More than 10 times a week	4	4.4
I do not consume chocolate	18	20
Q2/ Does consuming chocolate bring your headaches or make them worse?		
Yes	2	2.2
No	59	65.6
I don’t know	29	32.2
Dairy products		
Q1/ How often do you consume dairy products per week?		
1-3 times per week	45	50
5-10 times a week	29	32.2
More than 10 times a week	10	11.1
I do not consume chocolate	6	6.7
Q2/ Does consuming dairy bring your headaches or make them worse?		
Yes	0	0
No	60	66.7
I don’t know	30	33.3
Cheese		
Q1/ How often do you consume cheese per week?		
1-3 times per week	44	48.9
5-10 times a week	31	34.4
More than 10 times a week	6	6.7
I do not consume chocolate	9	10
Q2/ Does consuming cheese bring your headaches or make them worse?		
Yes	0	0
No	63	70
I don’t know	27	30
Caffeine		
Q1/ How often do you consume caffeine (coffee, tea, soft drinks) per week?		
1-3 times per week	22	24.4
5-10 times a week	36	40
More than 10 times a week	24	26.7
I do not consume chocolate	8	8.9
Q2/ Does consuming caffeine bring your headaches or make them worse?		
Yes	7	7.8

Table 12 shows the association between sleeping hours and headaches frequency and severity. No significant relationship was found between sleeping hours and headache frequency per month, headache severity (out of 10), and being awakened at night from headaches.

**Table 12:** The association between sleeping hours and headaches frequency and severity (n = 90).

Sleeping Hours	Headaches Frequency per Month (Mean + SD)			P-value
3-5 hours	5.73 + 6.1			0.816
6-8 hours	5.82 + 4.9			
More than 8 hours	4.43 + 0.98			
Sleeping hours	Headache pain severity (out of 10)			P-value
	Mild (1 - 3)	Moderate (4 - 6)	Severe (7 - 10)	
3-5 hours	3 (6.7%)	20 (44.4%)	22 (48.9%)	0.194
6-8 hours	5 (13.2%)	23 (60.5%)	10 (26.3%)	
More than 8 hours	0 (0%)	5 (71.4%)	2 (28.6%)	
Sleeping Hours	Do your headaches awaken you at night?			P-value
	Never	Occasionally	Often	
3-5 hours	26 (57.8%)	16 (35.6%)	3 (6.7%)	0.959
6-8 hours	23 (60.5%)	12 (31.6%)	3 (7.9%)	
More than 8 hours	4 (57.1%)	2 (28.6%)	1 (14.3%)	

## Discussion

The purpose of this study was to measure the prevalence of migraine among the female population of KSAU-HS - Jeddah and to assess whether the patients’ dietary habits had an impact on their headache frequency and severity. In this study, 410 subjects were interviewed and assessed using the ICHD-III migraine criteria. After statistically analyzing the data, it was concluded that migraine is prevalent among 40.24% of the female population at KSAU-HS.

This result differs significantly from what was reported by Muayqil et al. [2] about the prevalence of migraine among females in Saudi Arabia, which was found to be 20.1%. This difference may be attributed to the fact that all of our participants were either working women or students of health sciences. Comparably, Al Jumah et al. found that the prevalence of migraine is 36% among females in a population-based study in Saudi Arabia [11], which is close to our findings.

Variable prevalence among female students was reported in several other local studies. A higher prevalence was reported in a study done at Taibah University, which found the migraine prevalence to be 61.8% among female students [12]. A similar result (61.5%) was also reported in a study done at Jazan University [13]. On the other hand, two other studies in Taif University and the KSAU-HS Riyadh branch showed a lower prevalence of 32.5% and 5.30%, respectively [14, 15].

When comparing female students to employees, we found that the employees had a higher prevalence of migraine; 48.28% as compared to 38.92% of students. It was previously reported that migraine prevalence is the highest in females aged 25 to 55 years old, which is the peak of economic productivity, and several other studies have noticed that migraine is more prevalent among economically active individuals [16-18]. This may be attributed to increased responsibilities and work-related stressors.

The only food items reported to be triggers of migraine by participants were chocolate and caffeinated beverages. About 10% reported an association between the consumption of chocolate and caffeine. Only 2.2% reported that chocolate caused them to experience headaches, while 7.8% reported that caffeine caused them to develop headaches. Caffeinated beverages and chocolate are classically associated with migraine. However, the evidence is still not sufficient to make recommendations. A prospective study that evaluated the relationship between caffeine and migraine found that caffeine's effect is complex and follows a dose-dependent pattern. Consumption of high amounts of caffeine has been reported to trigger migraine headaches on the same day of consumption [19]. A recent review about chocolate as a trigger for migraine headaches evaluated 25 studies, and the conclusion was that there was no sufficient evidence to link chocolate consumption with the precipitation of migraine attacks. In some of the studies included, a small percentage of participants reported that chocolate was a trigger for their migraine headaches. However, these results can be questioned due to the study designs used and the possibility of recall bias [20]. This limitation also applies to the present study, as participants were asked to choose triggers from a list of food items. Also, by using a cross-sectional study design, we were unable to prove any associations.

Our study is not designed to discover new dietary triggers for migraine but to assess the hidden and neglected effects of the new trends in dietary cultures on migraine patients. The food industry with the unprecedented expansion in coffee shops needs to address dietary factors that may precipitate significant days of headache and loss of productivity in patients with migraine if calculated on an annual level. Public awareness is needed to highlight the neglected effects of coffee and tyramine-rich food on patients with migraine. Investing in dietary alternatives such as decaffeinated beverages or plant-based milk products may provide a range of new options for patients with migraine. Our study should prompt further impact assessment of the new trend in the coffee industry and its effect on health, quality of life, and economy on a nationwide level.

## Conclusions

Migraine remains a highly prevalent disorder among student populations with a significant impact on productivity and quality of life. Migraine may represent underrated social and economical burdens entailing absenteeism, excessive medical costs, and impaired quality of life. Our study revealed that the widespread trends for excessive consumption of coffee and caffeinated beverages at food outlets within the educational institution are occult triggers for headache attacks in a significant portion of students with migraine. The recent shift in dietary habits in our community for excessive consumption of coffee and other tyramine-rich food items has negative consequences on productivity and the economy. Our results can be conceivably extrapolated to reflect the effect of dietary habits on other streams of society, including companies, firms, schools, and workplaces that are consumed by the new dietary trends.
